# Butyrate-Induced Transcriptional Changes in Human Colonic Mucosa

**DOI:** 10.1371/journal.pone.0006759

**Published:** 2009-08-25

**Authors:** Steven A. L. W. Vanhoutvin, Freddy J. Troost, Henrike M. Hamer, Patrick J. Lindsey, Ger H. Koek, Daisy M. A. E. Jonkers, Andrea Kodde, Koen Venema, Robert J. M. Brummer

**Affiliations:** 1 TI Food and Nutrition, Wageningen, The Netherlands; 2 Division of Gastroenterology and Hepatology, Department of Internal Medicine, Maastricht University, Maastricht, The Netherlands; 3 Department of Population Genetics, Genomics and Bioinformatics, Maastricht University, Maastricht, The Netherlands; 4 Department of Bio Sciences, TNO Quality of life, Zeist, The Netherlands; 5 School of Health and Medical Sciences, Örebro University, Örebro, Sweden; Charité-Universitätsmedizin Berlin, Germany

## Abstract

**Background:**

Fermentation of dietary fiber in the colon results in the production of short chain fatty acids (mainly propionate, butyrate and acetate). Butyrate modulates a wide range of processes, but its mechanism of action is mostly unknown. This study aimed to determine the effects of butyrate on the transcriptional regulation of human colonic mucosa *in vivo*.

**Methodology/Principal Findings:**

Five hundred genes were found to be differentially expressed after a two week daily butyrate administration with enemas. Pathway analysis showed that the butyrate intervention mainly resulted in an increased transcriptional regulation of the pathways representing fatty acid oxidation, electron transport chain and oxidative stress. In addition, several genes associated with epithelial integrity and apoptosis, were found to be differentially expressed after the butyrate intervention.

**Conclusions/Significance:**

Colonic administration of butyrate in concentrations that can be achieved by consumption of a high-fiber diet enhances the maintenance of colonic homeostasis in healthy subjects, by regulating fatty acid metabolism, electron transport and oxidative stress pathways on the transcriptional level and provide for the first time, detailed molecular insight in the transcriptional response of gut mucosa to butyrate.

## Introduction

Short-chain fatty acids (SCFAs) are derived from the microbial fermentation of undigested dietary fibers in the colon. As micro-organisms preferably ferment carbohydrates, most saccharolytic fermentation occurs in the proximal colon. Depletion of carbohydrate sources in the distal colon leads to a switch from saccharolytic to proteolytic fermentation, which is less favorable due to the formation of potentially toxic products. Both these toxic products and the lower availability of SCFAs in the distal colon are hypothesized to be involved in the pathogenesis of gastro-intestinal disorders such as ulcerative colitis (UC) and cancer [1]–[3]. The amount of SCFAs (mainly acetate, propionate and butyrate) produced in the colon depends on the site of fermentation, the diet and the composition of the microbiota, and can account for up to 5–15% of the total energy requirements of humans [4]. Fecal concentrations of acetate, propionate and butyrate are found in a molar ratio of approximately 60∶20∶20 [5], [6], but limited data about luminal concentrations in specific parts of the colon are only available from sudden death patients. Due to rapid absorption and metabolism, actual concentrations may differ. Among the different SCFAs, butyrate is known to modulate numerous processes. It induces cell differentiation and strongly inhibits cell proliferation in tumor cell lines [7]–[13]. Colonocytes use butyrate as their primary energy source and in the absence of butyrate they undergo apoptosis, but opposite effects were seen in transformed cells, suggesting a possible anti-carcinogenic effect of butyrate [13]–[15]. Furthermore, butyrate may have an effect on inflammation [13], oxidative stress [13], intestinal barrier function [13], [16], [17], visceral perception and rectal compliance [18] and may play a role in satiety [19], [20].

Transcriptional responses of butyrate were studied mostly in cell lines [14], [15], [21]–[32] and some studies were performed in animals and human patients [3], [30], [33]–[35]. *In vitro* and animal studies showed that butyrate downregulates the expression of genes associated with proliferation and oxidative stress and upregulates the expression of Mucin associated genes (Muc 1–4), tight junction proteins (zonulin and occludin) and the butyrate transporter monocarboxylate transporter-1 (MCT1). In UC patients, butyrate was shown to increase the expression of the butyrate transporter MCT-1 and to decrease inflammation by inhibition of the activation of NF-κB. Effects of butyrate on global, genome-wide transcriptional responses of human intestinal mucosa were not described previously.

The aim of this study was to determine the *in vivo* genome-wide transcriptional response to local administration of butyrate in the distal colon in healthy volunteers in order to identify the biological processes mediated by butyrate, providing new leads for clinical and mechanistic studies.

## Materials and Methods

### Objectives

To determine the *in vivo* transcriptional response of a local administration of butyrate in the distal colon in healthy volunteers.

### Participants

Sixteen healthy volunteers (12 females and 4 males, 18 to 62yrs) participated in this study. Exclusion criteria were signs of bowel dysfunction, gastrointestinal surgery, age over 65 years, or use of any medication, probiotics or prebiotics three months prior to inclusion, were excluded from participation. All participants signed an informed consent prior to participation to the study, which was approved by the Ethical Committee of the University Hospital Maastricht, the Netherlands, and conducted in full accordance with the principles of the ‘Declaration of Helsinki’ (52^nd^ WMA General assembly, Edinburgh, Scotland, Oct 2000). The study has been registered in the US National Library of Medicine (http://www.clinicaltrials.gov) with reference code *NCT00693355.*

### Design

**Figure 1:**
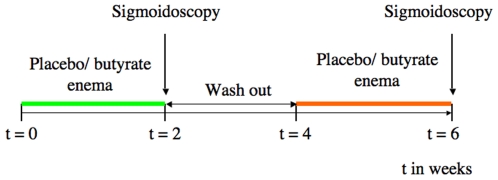
Design of butyrate study in healthy volunteers.

The study was performed according to a double-blind randomized placebo-controlled cross-over design. The protocol comprised of two experimental periods of two weeks each with a wash-out period of two weeks in between (**Figure 1**). During each experimental period, the subjects self-administered an enema containing 100 mM of butyrate or placebo (60 ml, pH 7.0), respectively, once daily prior to sleeping. The local hospital pharmacy department prepared all enemas. The volunteers were asked to defecate, if possible, prior to instillation of the enema. At the end of each experimental period, a sigmoidoscopy was performed in the morning after an overnight fast and biopsy samples were taken from a standardized location in the sigmoid colon (approx. 20 cm from the anal sphincter at the location of the internal iliac artery). All sigmoidoscopy procedures were performed in an unprepared colon to exclude possible effects induced by the colon cleansing procedure. The diet was standardized 3 days prior to the sigmoidoscopy. After sampling, biopsies were snap-frozen immediately in liquid nitrogen and stored at −80°C until further analysis.

### RNA extraction

RNA was isolated from frozen biopsies by adding a mixture of 1 ml Trizol (Invitrogen, Carlsbad, USA) and 10 µl β-mercaptoethanol, preheated up to 37°C. These mixtures were shaken for 30 seconds at maximum speed using a minibeadbeater. 200 µl Chloroform was added and after 3 minutes of incubation, the samples were centrifuged for 15 minutes, 21000 g at 4°C. 500 µl was taken from the upper colorless phase and mixed with 500 µl 70% ethanol. RNA was further purified with an RNeasy mini kit (Qiagen, Venlo, The Netherlands) combined with a DNase treatment using the RNase-Free DNase set (Qiagen, Venlo, The Netherlands) according to manufacturers protocol.

Quantity and purity of the RNA samples was determined using the Nanodrop ND-1000 spectrophotometer (Nanodrop Technologies, Wilmington, USA) and RNA integrity was determined using the Bioanalyzer 2100 (Agilent Technologies, Palo Alto, USA).

### Microarray hybridization

Total RNA (150 ng) was amplified using the two-cycle cDNA synthesis kit (Affymetrix, Santa Clara, USA) in combination with the MEGAscript T7 *in vitro* transcription system (Ambion). Double-stranded cDNA was biotin labeled with the GeneChip *in vitro* transcription IVT labeling kit (Affymetrix, Santa Clara, USA).

Following fragmentation, 11 µg of biotin-labeled cRNA were hybridized for 16 hour at 45°C on Affymetrix Human Genome U133 Plus 2.0 Arrays.

GeneChips were washed and stained in the Affymetrix Inc. Fluidics Station 450 (Affymetrix, Santa Clara, USA) and hybridized. Cyclic RNA was detected using streptavidin coupled to phycoerythrin. GeneChips were scanned using GeneChip Scanner 3000/7G and GeneChip Operating System (GCOS, Affymetrix, Santa Clara, USA) using Affymetrix default settings.

### Microarray analysis

Images of the Human Genome U133 Plus 2.0 arrays were quantified with GCOS software (Affymetrix). The chip description file (CDF) used for the analysis was an update created and freely distributed by the microarray lab of the university of Michigan (http://brainarray.mbni.med.umich.edu) [36] based on UniGenes (version 8). A more detailed description of this analysis is shown in the supplementary data (**Statistics S1**). Briefly, the genes were analyzed using a multivariate Gaussian linear regression including the hybridization and labeling spikes, the hybridization day, and a random effect to take into account multiple observations on the same subject. The inference criterion used for comparing the models is their ability to predict the observed data, i.e. models are compared directly through their minimized minus log-likelihood. When the numbers of parameters in models differ, they are penalized by adding the number of estimated parameters, a form of the Akaike Information Criterion (AIC) [37]. For each gene, the treatment group was then added to the model. The gene under consideration was found to be differentially expressed if the AIC decreased compared to the model not containing the treatment effect. Effects are considered significant if the 95% confidence intervals do not overlap. This analysis method avoids multiple testing issues and improves statistical power compared to the conventional approach.

### Pathway analysis

The genes analyzed and fold changes were loaded into GenMapp (http://genmapp.org) [38] and MAPPFinder [39] software packages to evaluate the transcripts in relation to known biological processes, molecular function and cellular component based on Gene Ontology (GO) terms [40] and contributed maps (i.e. local MAPPs). Only gene-transcripts with either their average intensities for the control and treated groups above 250 or average intensities for one of these groups above 500 and a 10 percent up or down regulation fold change were used to obtain a ranked list of pathways with differentially expressed genes.

MappFinder software was used to select the MAPPs with relatively high numbers of differentially expressed genes. The ranking of regulated pathways was indicated by the individual Z-scores. The Z-score increases when higher numbers of changing gene reporters relative to the number of genes on the MAPP are found on MAPPs. All pathways with both the Z-score and the number of genes changed>1 were considered to be significantly regulated. The results of the pathway analysis are presented in GO annotations **(Table S1)** and local MAPPs **(Table S2)**, which give a more precise representation of the biological pathways in which the measured genes are involved.

Transcriptional data are published in the public database “ArrayExpress” [41] under accession number E-MEXP-1705.

### Real-Time PCR

First strand cDNA was synthesized using the iScript cDNA Synthese kit (Bio-Rad, Veenendaal, The Netherlands) according to the manufacturer's instructions. 500 ng of the total RNA used for the microarray analysis was used as a template for the cDNA reaction. The cDNA was diluted with RNase free H_2_O to a concentration of 0.32 ng/µl. IQ Sybr Green Supermix (Bio-Rad, Veenendaal, The Netherlands) was used for the Q-PCR. Each Q-PCR reaction contained 12.5 µl iQ Sybr Green Supermix, 1 µl of 10 µM gene-specific forward and reverse primers, 4 µl diluted cDNA template and 6.5 µl sterile water. CANX, 18SrRNA and GAPDH were included as Housekeeping genes. Primers that were used are presented in **Table 1**.

**Table 1:** Genes that were selected for q-PCR analysis with the primers that were used.

Gene:	Sequence ID	Primer (forward, reverse):
18SrRNA	M10098	GTAACCCGTTGAACCCCATT, CCATCCAATCGGTAGTAGCG
GAPDH	NM_002046	TGCACCACCAACTGCTTAGC, GGCATGGACTGTGGTCATGAG
CANX	NM_001024649	CCACTGCTCCTCCTTCATCTCC, CGGTATCGTCTTTCTTGGCTTTGG
ACADM	NM_000016	GCCAGCGATGTTCAGATACTAGAGG, CCTTTCCAGGGCATACTTGGTAGC
GPX1	NM_000581	CCGACCCCAAGCTCATCACC, GATGTCAGGCTCGATGTCAATGG
GPX3	NM_002084	ACATGCCTACAGGTATGCGTGATTG, TGGAGTGGAGAACTGGAGAGAAAGG
GSR	NM_000637	CAGGGACTTGGGTGTGATGAAATGC, GAGGTAGGGTGAATGGCGACTGTG
NDUFA3	NM_004542	GGAGACAAAGATGGCTGCGAGAG, GTCAGTGGAGGTGCTCACAGTTTC
NDUFV1	NM_007103	GCCATCGCCCGCCTCATTG, CCGTCACCCAGAGCACAAATCG

Q-PCR reactions were run on the My IQ Single Color Real Time PCR Detection System (Bio-Rad, Veenendaal, The Netherlands). After 3 minutes of incubation at 95°C, an amplification cycle program of 40 cycles of 10 seconds at 95°C and 45 seconds at 60°C, followed by a melting program was initiated.

## Results

### Micro-array analysis

We used microarray analysis to assess the expression levels of all human genes in colonic biopsies after butyrate treatment compared to placebo. In total, 501 genes were found to be differentially expressed after the butyrate intervention compared to placebo (**Table S3**). From those genes, 473 were up regulated and 28 down regulated.

### Pathway analysis

**Figure 2:**
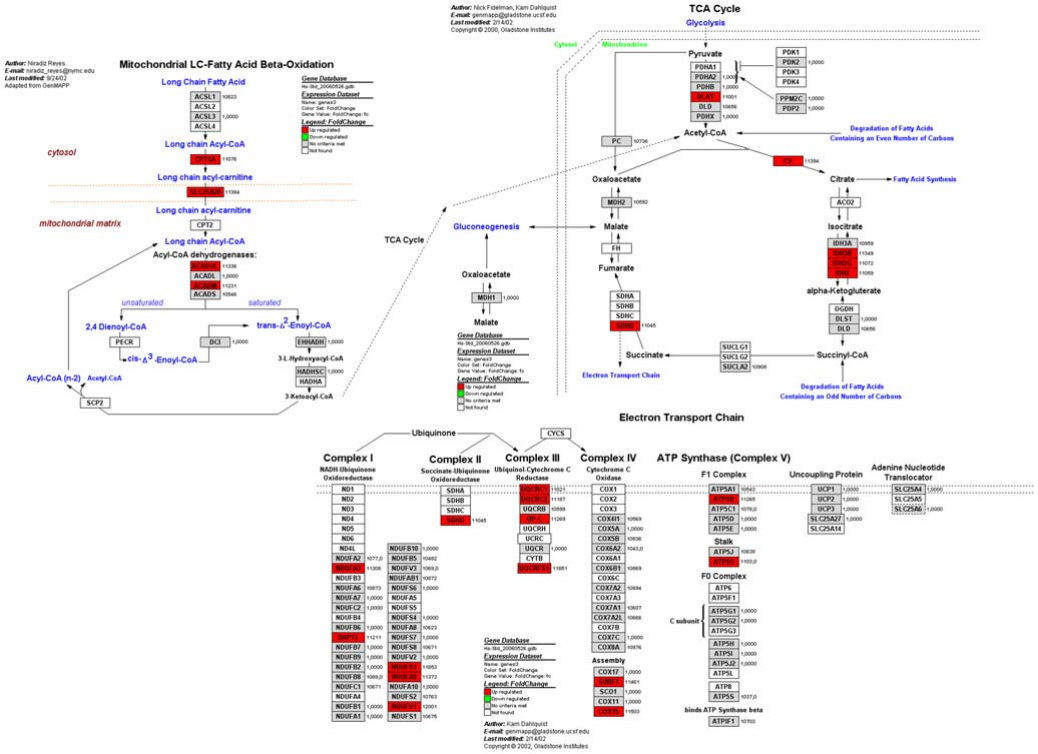
Differentially expressed genes in Pathways for fatty acid metabolism, TCA cycle and the electron transport chain. This Figure was merged from 3 individual Local Mapps in Genmapp.

Pathway analysis with Genmapp software delivered a list of significantly regulated pathways, ranked by z-score (**Table S1 and S2**). The butyrate intervention mainly regulated the citric acid cycle (TCA-cycle) (**Figure 2**), fatty acid transport and oxidation, electron transport (**Figure 2**), TNF-alpha signaling and oxidative stress related pathways. In the TCA cycle pathway, citrate synthase and some genes involved in the formation of α-ketogluterate out of isocitrate were upregulated. In the pathway of fatty acid metabolism, genes for transport and oxidation of medium and long chain fatty acids were expressed differentially. The pathway analyses also showed a number of differentially expressed genes in the electron transport chain (**Figure 2**). Most of these genes (9 out of 14) were present in Complex I and III of the electron transport chain. In the oxidative stress pathway, a number of genes involved in glutathione metabolism (GPX1, GPX3 and GSR) were differentially expressed.

### Validation of microarray analysis by q-PCR

Based on microarray- and pathway analyses, 6 genes of interest were selected for confirmation by q-PCR. The increased expression of Acadm, Gpx3, Gpx1 and Ndufa3, identified by the micro array analysis, was confirmed by q-PCR (**Table 2**). The gene reporters Ndufv1 and Gsr were upregulated in the micro array, but downregulated in the q-PCR analysis.

**Table 2:** Fold changes of q-PCR and microarray analysis for 7 genes of interest.

Gene	Micro-array (fc)	q-PCR (fc)
Acadm	1.12	1.11
Gpx3	1.25	1.15
Gpx1	1.13	1.07
Ndufv1	1.20	0.91
Ndufa3	1.13	1.13
Gsr	1.11	0.93

## Discussion

Local administration of butyrate in the distal colon resulted in an increased transcription of genes, which were mainly associated with energy metabolism, fatty acid metabolism and oxidative stress. These results are in line with effects reported in literature, as reviewed recently by our group [13]. We showed for the first time that these processes are significantly regulated on the transcriptional level by intraluminal butyrate in healthy humans.

The impact of the butyrate administration as presented in this study with effects on gene transcription up to 39%, was lower compared to previous findings in cell lines and animal studies, probably due to the fact that butyrate was studied in healthy volunteers in the most physiologically achievable way. Studying humans *in vivo* gives a larger variance in study data due to limitations of standardization as well as the genomic variability compared to animal and *in vitro* studies. In contrast to stress models in animals and patients suffering from gastrointestinal disorders, healthy volunteers do not have a compromised gut. The beneficial effects that can be expected from the present intervention are, consequently, small compared to a compromised situation like in animals, cell lines or patients. The concentration of butyrate used in the present study (100 mM) was physiologically achievable by consuming a high fiber diet, in contrast to a pharmacological dose as used in some previous studies.

The microarray data show that fatty acid metabolism is regulated by butyrate, as a number of genes associated with processes involved in fatty acid transport, primary steps of beta oxidation and the formation of keton bodies were regulated. The transcription of genes encoding the fatty acid transporters carnitine palmityl-CoA transferase 1 (CPT1) and carnitine-acylcarnitine translocase (SLC25A20) was increased. CPT1 is located in the outer mitochondrial membrane and promotes the transport of long chain fatty acids into the mitochondrion by binding carnitine to the fatty acids [42], [43]. Transport of carnitine-linked long chain fatty acids over the inner mitochondrial membrane is facilitated by SLC25A20 in exchange for free carnitine [44], [45]. These two genes promote long chain fatty acid transport from the cytosol to the mitochondrial matrix where β-oxidation starts.

The first step of β-oxidation is the formation of 2-eonyl-CoA from the corresponding saturated ester, catalyzed by SLC25A20. For dehydrogenation of acyl-CoA, 4 enzymes are described, each targeting fatty acids of a specific chain length: short-chain-acyl-CoA dehydrogenase (ACADS, with C4 and C6 specificity), medium- chain-acyl-CoA dehydrogenase (ACADM, with C4-C12 specificity), long-chain-acyl-CoA dehydrogenase (ACADL, active with C8-C20) and very-long-chain-acyl-CoA dehydrogenase (ACADVL, active with C12-C24) [43]. The butyrate (a C4 fatty acid) intervention resulted in an increased expression of both ACADM (confirmed with q-PCR), located in the mitochondrial matrix, and ACADVL, which is situated in the inner mitochondrial membrane. The intervention did not clearly modulate the transcriptional regulation of ACADS, in spite of its activity on C4-fatty acids. Next to mediating fatty acid transport, the rate of mitochondrial β-oxidation may also be limited by an accumulation of acetyl-CoA. This can be prevented by the observed increased transcription of both citrate synthase (CS), which drives the citric acid cycle, and by mitochondrial 3-hydroxy-3-methylglutaryl-CoA synthase (HMGCS2). HMGCS2 converts acetyl-CoA to ketone bodies [46] thereby preventing the accumulation of acetyl-CoA [47]. In humans, HMGCS2 is expressed in liver, skeletal muscle, heart, pancreas, testis and colon[48]. In rats, the expression of HMGCS2 in the colon depends on the amount of butyrate produced by the intestinal microbiota [49], [50]. The mediation of fatty acid transport and HMGCS2 together with the increased ACADM and ACADVL expression suggests that butyrate is able to regulate the rate of fatty acid oxidation.

Butyrate is known to inhibit proliferation in colonic tumor cells and cell lines [7], [12] but to stimulate proliferation in healthy colonic epithelial cells [51], [52]. This is often referred to as “the butyrate paradox” [7]. It was suggested previously that HMCGS2 is involved in the inhibiting effect of butyrate on cell proliferation [53]. HMGCS2 expression in colonic epithelial cells is butyrate dependent and correlates with the capacity of the colon for ketogenesis and the fatty acid oxidation rate [49], [50]. One explanation for the butyrate paradox is that healthy cells have an efficient butyrate metabolism resulting in low intracellular butyrate concentrations and therefore a decrease in capacity to inhibit growth [53]. In colon cancer cell lines, β-oxidation and HMGCS2 expression are impaired [53]. The decreased oxidation rate of butyrate may result in increased intra-cellular butyrate concentrations in tumor cells, hence causing increased histone deacetylation and subsequently decreased proliferation. The observation that butyrate affects proliferation is strengthened by the finding in the present study that several genes which are known to be involved in either proliferation, cell growth or cell size were differentially expressed by the butyrate intervention.

Butyrate mediated the transcription of genes that are involved in pyruvate dehydrogenase, citric acid cycle and the respiratory chain. The gene dihydrolipoamide acetyltransferase (DLAT), which encodes for a subunit of the pyruvate dehydrogenase complex forming acetyl-CoA from pyruvate was upregulated. Butyrate also increased the transcription of the genes that encode citrate synthase (CS) and succinate dehydrogenase (SDHD). Citrate synthase is the first enzyme of the TCA- cycle and catalyses the condensation of oxaloacetate, a cyclic acid cycle intermediate, and acetyl-CoA to form citrate. SDHD, which is also directly coupled to complex two of the electron transport chain, oxidizes succinate to fumarate as the first part of the final step of the citric acid cycle [54]. Nicotinamide adenine dinucleotide (NADH) and flavin adenine dinucleotide (FADH_2_), formed by glycolysis, the TCA- cycle and β-oxidation, subsequently enter the electron transport chain where the electrons are transferred along the respiratory chain in order to form ATP. Butyrate induced increased transcription of genes participating in all five complexes of the respiratory chain. In complex one, the genes NDUFA3 and NDUFV1 were significantly upregulated (and confirmed by q-PCR). SDHD, active in complex two of the respiratory chain, was upregulated. In total, 14 out of 72 genes that participate in the respiratory chain were upregulated (**Table S2**). In conclusion, butyrate regulated numerous genes involved in all parts of the total energy metabolism and ATP production, which provides molecular support for the general assumption that butyrate is involved in the energy metabolism of colonic epithelial cells.

Oxidative stress is an imbalance between the anti-oxidant defense network and the production of reactive oxygen species (ROS) and reactive nitrogen species (RNS). Butyrate has previously been shown to affect oxidative stress and inflammation [13], [55].

In the present study, oxidative stress related pathways and NF-κB signaling were shown to be affected by butyrate. More specifically, the expression of glutathione peroxidases GPX 1 and GPX 3 and glutathione reductase (GSR) were upregulated in the oxidative stress pathway.

Glutathione (GSH) is used to eliminate reactive oxygen species, a reaction that is catalyzed by glutathione peroxidase. The glutathione disulfide (GSSG) produced in this reaction can be converted back to GSH by the action of the enzyme glutathione reductase (GSR)[56]. In the present study, the increased expression of GPX1, GPX3 and GSR suggests that butyrate induces an increased glutathione turnover capacity and an increased antioxidant capacity. This was in line with earlier findings from our group showing an increased GSH production after butyrate administration [55]. GSH can also detoxify harmful electrophils, and is catalyzed by glutathione-S-transferase (GST). Previous studies showed that butyrate induced the expression levels of GST [57], [58]. However, we did not observe transcriptional regulation of GST in the present study. The previously reported finding that butyrate mediates the JAK/STAT signaling pathway [59], which plays an important role in the regulation of NO production in epithelial cells, supports the upregulation of one of the other genes in the oxidative stress pathway in the present study, NADH quinone oxidoreductase 1(NQO1), which also mediates nitric oxide (NO) biosynthesis. The current observation suggests that butyrate may increase stimulation of epithelial proliferation, migration and apoptosis.

In an inflamed colon, reactive oxygen species (ROS) are produced by neutrophilic granulocytes, which are associated with increased oxidative stress as was previously reported in ulcerative colitis and Crohn's disease [60], [61]. In the present study, the gene encoding nuclear factor kappa beta inhibitor α (NFKBIA), was upregulated. NFKBIA inhibits the activation of NF-κB and the TNF-α signaling cascade, thereby potentially leading to diminished inflammation and inflammation-induced oxidative stress.

Another important biological process affected by butyrate is proteasome degradation. This process, in which eight genes were differentially expressed (**Table S2**), provides a mechanism for degradation of (oxidatively) damaged proteins and the genes in this pathway are associated with apoptosis, ageing and oxidative stress [62].

Hence, butyrate regulated genes that are associated with glutathione metabolism, inflammation, NO synthesis and proteasome degradation, all supporting its previously demonstrated potential to reduce inflammation and oxidative stress.

The q-PCR analyses confirmed the microarray results of 4 out of 6 genes confirmed the microarray results, whereas two genes were upregulated in the microarray analysis while they were downregulated in the q-PCR analysis. One gene was not significantly regulated based on the microarray results but showed a downregulation in the q-PCR analysis. There is international consensus that the Affymetrix microarray technology provides a reliable platform to measure gene expression [63]. The observed differences between microarray and q-PCR analysis are in line with earlier observations and can also be explained by differences in probe sequence and thus target location [63]. Because of these known problems to confirm microarray data with q-PCR, the relevance and importance of such a comparison should be reconsidered. Pathway analysis in combination with stringent statistical methods provides a strong indication for the quality of the microarray measurement. If pathway analysis results in significantly regulated pathways, a number of genes cluster within the same process, hence increasing the likelihood that the fold changes of these individual genes, measured simultaneously, were correct. The added value of the combination of both microarray analysis and the pathway analysis should be point of discussion when evaluating the reliability of the outcome of the microarray analysis.

Butyrate was administered by rectal enemas, because this is a safe and reliable way to deliver a specific amount of substrate to the distal colon. Other techniques, such as oral intake of dietary fibers or encapsulated butyrate, do not allow to accurately target the distal colon *in vivo*. The distal colon was chosen as target area for the butyrate intervention since the concentration of butyrate is lowest in this part of the colon due to rapid fermentation of commonly ingested dietary fibers in the proximal colon and the incidence of carcinomas and diseases in particularly the distal part of the colon is rising [1], [2]. Furthermore, a mucosal specimen of the distal colon can be obtained much more easily compared to that of the proximal colon, without sedation and previous bowel cleansing. The latter is of pivotal importance in this type of studies to avoid disturbance of the physiological conditions in the gut, leading to interference with the effects of the intervention. Volunteers instilled the enemas in the evening prior to sleeping in order to obtain an optimal spread of the butyrate in the distal colon and to minimize the risk of leakage. The spread of enemas was studied previously in patients with ulcerative colitis [64]–[67]. In a pilot study in healthy volunteers, we confirmed, using enemas containing radio actively labeled Indium, that the spread of the 60 ml enemas used in the present study reached beyond the sigmoid colon [unpublished data]. More research is needed on the actual concentration of butyrate at different locations in the human colon *in vivo* to optimize the dose and method of administration.

This study showed for the first time in healthy volunteers the effect of butyrate treatment on the gene transcriptional level in the distal colon. Previously observed beneficial effects of butyrate from patient, animal- and *in vitro* studies are also induced in healthy subjects. The results presented in this study provide new leads to study the mechanisms involved in the effects of butyrate in humans. Future studies should be planned in order to study and optimize the functional consequences of butyrate or dietary fiber in the colon.

## Supporting Information

Table S1 This table shows the GO-annotations, ranked by Z-score
(0.15 MB DOC)

Table S2 This table shows the local mapps, ranked by Z-score
(0.07 MB DOC)

Table S3 This Table shows each of the 501 significantly regulated genes and the foldchanges.
(0.07 MB XLS)

Statistics S1 This supporting information describes in more detail, the statistics that were performed on the data
(0.03 MB DOC)

## References

[1] Marteau P (2006). Probiotics, prebiotics, synbiotics: ecological treatment for inflammatory bowel disease?. Gut.

[2] Le Leu RK, Brown IL, Hu Y, Morita T, Esterman A (2007). Effect of dietary resistant starch and protein on colonic fermentation and intestinal tumourigenesis in rats.. Carcinogenesis.

[3] Wong CS, Sengupta S, Tjandra JJ, Gibson PR (2005). The influence of specific luminal factors on the colonic epithelium: high-dose butyrate and physical changes suppress early carcinogenic events in rats.. Dis Colon Rectum.

[4] Bergman EN (1990). Energy contributions of volatile fatty acids from the gastrointestinal tract in various species.. Physiol Rev.

[5] Topping DL, Clifton PM (2001). Short-chain fatty acids and human colonic function: roles of resistant starch and nonstarch polysaccharides.. Physiol Rev.

[6] Hallert C, Bjorck I, Nyman M, Pousette A, Granno C (2003). Increasing fecal butyrate in ulcerative colitis patients by diet: controlled pilot study.. Inflamm Bowel Dis.

[7] Comalada M, Bailon E, de Haro O, Lara-Villoslada F, Xaus J (2006). The effects of short-chain fatty acids on colon epithelial proliferation and survival depend on the cellular phenotype.. J Cancer Res Clin Oncol.

[8] Ranganna K, Joshi T, Yatsu FM (1995). Sodium butyrate inhibits platelet-derived growth factor-induced proliferation of vascular smooth muscle cells.. Arterioscler Thromb Vasc Biol.

[9] Ranganna K, Yatsu FM, Hayes BE, Milton SG, Jayakumar A (2000). Butyrate inhibits proliferation-induced proliferating cell nuclear antigen expression (PCNA) in rat vascular smooth muscle cells.. Mol Cell Biochem.

[10] Prasad KN (1980). Butyric acid: a small fatty acid with diverse biological functions.. Life Sci.

[11] Hague A, Butt AJ, Paraskeva C (1996). The role of butyrate in human colonic epithelial cells: an energy source or inducer of differentiation and apoptosis?. Proc Nutr Soc.

[12] Hodin RA, Meng S, Archer S, Tang R (1996). Cellular growth state differentially regulates enterocyte gene expression in butyrate-treated HT-29 cells.. Cell Growth Differ.

[13] Hamer HM, Jonkers D, Venema K, Vanhoutvin S, Troost FJ (2008). Review article: the role of butyrate on colonic function.. Aliment Pharmacol Ther.

[14] Cuff M, Dyer J, Jones M, Shirazi-Beechey S (2005). The human colonic monocarboxylate transporter Isoform 1: its potential importance to colonic tissue homeostasis.. Gastroenterology.

[15] Pajak B, Orzechowski A, Gajkowska B (2007). Molecular basis of sodium butyrate-dependent proapoptotic activity in cancer cells.. Adv Med Sci.

[16] Mariadason JM, Kilias D, Catto-Smith A, Gibson PR (1999). Effect of butyrate on paracellular permeability in rat distal colonic mucosa ex vivo.. J Gastroenterol Hepatol.

[17] Peng L, He Z, Chen W, Holzman IR, Lin J (2007). Effects of butyrate on intestinal barrier function in a Caco-2 cell monolayer model of intestinal barrier.. Pediatr Res.

[18] Vanhoutvin SA, Troost FJ, Kilkens TO, Lindsey PJ, Hamer HM (2009). The effects of butyrate enemas on visceral perception in healthy volunteers.. Neurogastroenterol Motil.

[19] Cherbut C (2003). Motor effects of short-chain fatty acids and lactate in the gastrointestinal tract.. Proc Nutr Soc.

[20] Cani PD, Joly E, Horsmans Y, Delzenne NM (2006). Oligofructose promotes satiety in healthy human: a pilot study.. Eur J Clin Nutr.

[21] Daly K, Shirazi-Beechey SP (2006). Microarray analysis of butyrate regulated genes in colonic epithelial cells.. DNA Cell Biol.

[22] Li RW, Li C (2006). Butyrate induces profound changes in gene expression related to multiple signal pathways in bovine kidney epithelial cells.. BMC Genomics.

[23] Daly K, Cuff MA, Fung F, Shirazi-Beechey SP (2005). The importance of colonic butyrate transport to the regulation of genes associated with colonic tissue homoeostasis.. Biochem Soc Trans.

[24] Li CJ, Li RW, Wang YH, Elsasser TH (2007). Pathway analysis identifies perturbation of genetic networks induced by butyrate in a bovine kidney epithelial cell line.. Funct Integr Genomics.

[25] Ranganna K, Yousefipour Z, Yatsu FM, Milton SG, Hayes BE (2003). Gene expression profile of butyrate-inhibited vascular smooth muscle cell proliferation.. Mol Cell Biochem.

[26] Tabuchi Y, Takasaki I, Doi T, Ishii Y, Sakai H (2006). Genetic networks responsive to sodium butyrate in colonic epithelial cells.. FEBS Lett.

[27] Joseph J, Mudduluru G, Antony S, Vashistha S, Ajitkumar P (2004). Expression profiling of sodium butyrate (NaB)-treated cells: identification of regulation of genes related to cytokine signaling and cancer metastasis by NaB.. Oncogene.

[28] Mariadason JM, Corner GA, Augenlicht LH (2000). Genetic reprogramming in pathways of colonic cell maturation induced by short chain fatty acids: comparison with trichostatin A, sulindac, and curcumin and implications for chemoprevention of colon cancer.. Cancer Res.

[29] Miller SJ, Zaloga GP, Hoggatt AM, Labarrere C, Faulk WP (2005). Short-chain fatty acids modulate gene expression for vascular endothelial cell adhesion molecules.. Nutrition.

[30] Thibault R, De Coppet P, Daly K, Bourreille A, Cuff M (2007). Down-regulation of the monocarboxylate transporter 1 is involved in butyrate deficiency during intestinal inflammation.. Gastroenterology.

[31] Williams EA, Coxhead JM, Mathers JC (2003). Anti-cancer effects of butyrate: use of micro-array technology to investigate mechanisms.. Proc Nutr Soc.

[32] Bordin M, D'Atri F, Guillemot L, Citi S (2004). Histone deacetylase inhibitors up-regulate the expression of tight junction proteins.. Mol Cancer Res.

[33] Kien CL, Blauwiekel R, Bunn JY, Jetton TL, Frankel WL (2007). Cecal infusion of butyrate increases intestinal cell proliferation in piglets.. J Nutr.

[34] Kien CL, Peltier CP, Mandal S, Davie JR, Blauwiekel R (2008). Effects of the in vivo supply of butyrate on histone acetylation of cecum in piglets.. JPEN J Parenter Enteral Nutr.

[35] Gaudier E, Rival M, Buisine M, Robineau I, Hoebler C (2008). Butyrate enemas upregulate Muc genes expression but decrease adherent mucus thickness in mice colon.. Physiol Res.

[36] Dai M, Wang P, Boyd AD, Kostov G, Athey B (2005). Evolving gene/transcript definitions significantly alter the interpretation of GeneChip data.. Nucleic Acids Res.

[37] Akaike H (1973). Information theory and an extension of the maximum likelihood principle.. Second international symposium on inference theory (Tsahkadsor,1971).

[38] Dahlquist KD, Salomonis N, Vranizan K, Lawlor SC, Conklin BR (2002). GenMAPP, a new tool for viewing and analyzing microarray data on biological pathways.. Nat Genet.

[39] Doniger SW, Salomonis N, Dahlquist KD, Vranizan K, Lawlor SC (2003). MAPPFinder: using Gene Ontology and GenMAPP to create a global gene-expression profile from microarray data.. Genome Biol.

[40] Ashburner M, Ball CA, Blake JA, Botstein D, Butler H (2000). Gene ontology: tool for the unification of biology. The Gene Ontology Consortium.. Nat Genet.

[41] Parkinson H, Kapushesky M, Kolesnikov N, Rustici G, Shojatalab M (2009). ArrayExpress update—from an archive of functional genomics experiments to the atlas of gene expression.. Nucleic Acids Res.

[42] Gregersen N, Andresen BS, Corydon MJ, Corydon TJ, Olsen RK (2001). Mutation analysis in mitochondrial fatty acid oxidation defects: Exemplified by acyl-CoA dehydrogenase deficiencies, with special focus on genotype-phenotype relationship.. Hum Mutat.

[43] Eaton S, Bartlett K, Pourfarzam M (1996). Mammalian mitochondrial beta-oxidation.. Biochem J.

[44] Korman SH, Pitt JJ, Boneh A, Dweikat I, Zater M (2006). A novel SLC25A20 splicing mutation in patients of different ethnic origin with neonatally lethal carnitine-acylcarnitine translocase (CACT) deficiency.. Mol Genet Metab.

[45] Indiveri C, Iacobazzi V, Giangregorio N, Palmieri F (1997). The mitochondrial carnitine carrier protein: cDNA cloning, primary structure and comparison with other mitochondrial transport proteins.. Biochem J.

[46] Dashti N, Ontko JA (1979). Rate-limiting function of 3-hydroxy-3-methylglutaryl-coenzyme A synthase in ketogenesis.. Biochem Med.

[47] Le May C, Pineau T, Bigot K, Kohl C, Girard J (2000). Reduced hepatic fatty acid oxidation in fasting PPARalpha null mice is due to impaired mitochondrial hydroxymethylglutaryl-CoA synthase gene expression.. FEBS Lett.

[48] Mascaro C, Buesa C, Ortiz JA, Haro D, Hegardt FG (1995). Molecular cloning and tissue expression of human mitochondrial 3-hydroxy-3-methylglutaryl-CoA synthase.. Arch Biochem Biophys.

[49] Cherbuy C, Darcy-Vrillon B, Morel MT, Pegorier JP, Duee PH (1995). Effect of germfree state on the capacities of isolated rat colonocytes to metabolize n-butyrate, glucose, and glutamine.. Gastroenterology.

[50] Cherbuy C, Andrieux C, Honvo-Houeto E, Thomas M, Ide C (2004). Expression of mitochondrial HMGCoA synthase and glutaminase in the colonic mucosa is modulated by bacterial species.. Eur J Biochem.

[51] Mortensen FV, Langkilde NC, Joergensen JC, Hessov I (1999). Short-chain fatty acids stimulate mucosal cell proliferation in the closed human rectum after Hartmann's procedure.. Int J Colorectal Dis.

[52] Scheppach W, Bartram P, Richter A, Richter F, Liepold H (1992). Effect of short-chain fatty acids on the human colonic mucosa in vitro.. JPEN J Parenter Enteral Nutr.

[53] Camarero N, Mascaro C, Mayordomo C, Vilardell F, Haro D (2006). Ketogenic HMGCS2 Is a c-Myc target gene expressed in differentiated cells of human colonic epithelium and down-regulated in colon cancer.. Mol Cancer Res.

[54] Stryer L (1997). Biochemistry: W.H. Freeman and Company New York..

[55] Hamer HM, Jonkers DM, Bast A, Vanhoutvin SA, Fischer MA (2009). Butyrate modulates oxidative stress in the colonic mucosa of healthy humans.. Clin Nutr.

[56] Maher P (2005). The effects of stress and aging on glutathione metabolism.. Ageing Res Rev.

[57] Pool-Zobel BL, Selvaraju V, Sauer J, Kautenburger T, Kiefer J (2005). Butyrate may enhance toxicological defence in primary, adenoma and tumor human colon cells by favourably modulating expression of glutathione S-transferases genes, an approach in nutrigenomics.. Carcinogenesis.

[58] Ebert MN, Klinder A, Peters WH, Schaferhenrich A, Sendt W (2003). Expression of glutathione S-transferases (GSTs) in human colon cells and inducibility of GSTM2 by butyrate.. Carcinogenesis.

[59] Stempelj M, Kedinger M, Augenlicht L, Klampfer L (2007). Essential role of the JAK/STAT1 signaling pathway in the expression of inducible nitric-oxide synthase in intestinal epithelial cells and its regulation by butyrate.. J Biol Chem.

[60] Kruidenier L, Verspaget HW (2002). Review article: oxidative stress as a pathogenic factor in inflammatory bowel disease—radicals or ridiculous?. Aliment Pharmacol Ther.

[61] Kruidenier L, Kuiper I, Lamers CB, Verspaget HW (2003). Intestinal oxidative damage in inflammatory bowel disease: semi-quantification, localization, and association with mucosal antioxidants.. J Pathol.

[62] Glickman MH, Ciechanover A (2002). The ubiquitin-proteasome proteolytic pathway: destruction for the sake of construction.. Physiol Rev.

[63] Canales RD, Luo Y, Willey JC, Austermiller B, Barbacioru CC (2006). Evaluation of DNA microarray results with quantitative gene expression platforms.. Nat Biotechnol.

[64] Campieri M, Corbelli C, Gionchetti P, Brignola C, Belluzzi A (1992). Spread and distribution of 5-ASA colonic foam and 5-ASA enema in patients with ulcerative colitis.. Dig Dis Sci.

[65] Chapman NJ, Brown ML, Phillips SF, Tremaine WJ, Schroeder KW (1992). Distribution of mesalamine enemas in patients with active distal ulcerative colitis.. Mayo Clin Proc.

[66] Kruis W, Bull U, Eisenburg J, Paumgartner G (1982). Retrograde colonic spread of sulphasalazine enemas.. Scand J Gastroenterol.

[67] Vitti RA, Meyers F, Knight LC, Siegel JA, Malmud LS (1989). Quantitative distribution of radiolabeled 5-aminosalicylic acid enemas in patients with left-sided ulcerative colitis.. Dig Dis Sci.

