# Supplementary material for: Butyrate-Induced Transcriptional Changes in Human Colonic Mucosa
Source: PLoS One. 2009 Aug 25;4(8):e6759. doi: 10.1371/journal.pone.0006759 (PMC2727000; doi:10.1371/journal.pone.0006759)
Supplement: Table S1 — This table shows the GO-annotations, ranked by Z-score (0.15 MB DOC) [file pone.0006759.s001.doc]

S1: GO-annotations, ranked by Z-score

| GOID | GO Name | Number Changed | Number Measured | Number in GO | Z Score |
| --- | --- | --- | --- | --- | --- |
| 8180 | signalosome complex | 2 | 2 | 9 | 9,299 |
| 4128 | cytochrome-b5 reductase activity | 2 | 2 | 5 | 9,299 |
| 5833 | hemoglobin complex | 2 | 3 | 9 | 7,505 |
| 3756 | protein disulfide isomerase activity | 2 | 3 | 9 | 7,505 |
| 5737 | cytoplasm | 58 | 1123 | 3067 | 7,269 |
| 30126 | COPI vesicle coat | 2 | 4 | 8 | 6,424 |
| 6672 | ceramide metabolism | 3 | 9 | 16 | 6,275 |
| 3735 | structural constituent of ribosome | 6 | 33 | 309 | 6,169 |
| 5739 | mitochondrion | 15 | 176 | 598 | 5,672 |
| 5344 | oxygen transporter activity | 2 | 6 | 16 | 5,122 |
| 6809 | nitric oxide biosynthesis | 2 | 6 | 15 | 5,122 |
| 6635 | fatty acid beta-oxidation | 2 | 6 | 13 | 5,122 |
| 6890 | retrograde vesicle-mediated transport\, Golgi to ER | 2 | 7 | 13 | 4,685 |
| 15671 | oxygen transport | 2 | 7 | 17 | 4,685 |
| 5840 | ribosome | 4 | 26 | 283 | 4,511 |
| 8430 | selenium binding | 2 | 8 | 29 | 4,329 |
| 6891 | intra-Golgi vesicle-mediated transport | 2 | 8 | 21 | 4,329 |
| 8601 | protein phosphatase type 2A regulator activity | 2 | 9 | 17 | 4,031 |
| 9306 | protein secretion | 2 | 10 | 23 | 3,776 |
| 6909 | phagocytosis | 2 | 10 | 22 | 3,776 |
| 5622 | intracellular | 84 | 2775 | 7205 | 3,721 |
| 5783 | endoplasmic reticulum | 11 | 172 | 474 | 3,701 |
| 6118 | electron transport | 9 | 127 | 366 | 3,697 |
| 16192 | vesicle-mediated transport | 10 | 150 | 335 | 3,676 |
| 3824 | catalytic activity | 63 | 1916 | 4973 | 3,675 |
| 5768 | endosome | 3 | 22 | 60 | 3,595 |
| 16853 | isomerase activity | 4 | 36 | 135 | 3,582 |
| 19825 | oxygen binding | 2 | 11 | 35 | 3,555 |
| 6629 | lipid metabolism | 13 | 230 | 570 | 3,528 |
| 8152 | metabolism | 82 | 2799 | 7383 | 3,274 |
| 6457 | protein folding | 6 | 80 | 228 | 3,173 |
| 6412 | protein biosynthesis | 10 | 185 | 644 | 2,922 |
| 16491 | oxidoreductase activity | 11 | 213 | 655 | 2,902 |
| 8021 | synaptic vesicle | 2 | 15 | 46 | 2,888 |
| 6810 | transport | 34 | 974 | 2446 | 2,823 |
| 30125 | clathrin vesicle coat | 2 | 16 | 36 | 2,758 |
| 16740 | transferase activity | 24 | 639 | 1575 | 2,69 |
| 6888 | ER to Golgi vesicle-mediated transport | 2 | 18 | 62 | 2,529 |
| 8757 | S-adenosylmethionine-dependent methyltransferase activity | 3 | 35 | 85 | 2,518 |
| 8415 | acyltransferase activity | 4 | 56 | 122 | 2,469 |
| 6631 | fatty acid metabolism | 4 | 57 | 139 | 2,427 |
| 51082 | unfolded protein binding | 4 | 66 | 159 | 2,088 |
| 5975 | carbohydrate metabolism | 8 | 184 | 451 | 1,934 |
| 6886 | intracellular protein transport | 6 | 128 | 365 | 1,867 |
| 8380 | RNA splicing | 3 | 48 | 156 | 1,866 |
| 1558 | regulation of cell growth | 3 | 49 | 108 | 1,826 |
| 6310 | DNA recombination | 2 | 28 | 99 | 1,742 |
| 5215 | transporter activity | 17 | 520 | 1265 | 1,619 |
| 20037 | heme binding | 2 | 31 | 87 | 1,573 |
| 5625 | soluble fraction | 4 | 85 | 197 | 1,527 |
| 8285 | negative regulation of cell proliferation | 3 | 58 | 140 | 1,499 |
| 6470 | protein amino acid dephosphorylation | 3 | 59 | 131 | 1,466 |
| 16829 | lyase activity | 3 | 60 | 149 | 1,434 |
| 15031 | protein transport | 7 | 193 | 541 | 1,297 |
| 5489 | electron transporter activity | 3 | 66 | 180 | 1,255 |
| 139 | Golgi membrane | 2 | 38 | 82 | 1,249 |
| 16757 | transferase activity\, transferring glycosyl groups | 4 | 97 | 224 | 1,244 |
| 3714 | transcription corepressor activity | 2 | 39 | 88 | 1,208 |
| 4295 | trypsin activity | 2 | 39 | 111 | 1,208 |
| 6508 | proteolysis | 8 | 245 | 608 | 1,08 |
| 74 | regulation of progression through cell cycle | 6 | 175 | 424 | 1,054 |
| 9117 | nucleotide metabolism | 3 | 74 | 197 | 1,044 |
| 8283 | cell proliferation | 7 | 213 | 503 | 1,025 |
| 4 | biological process unknown | 8 | 250 | 705 | 1,02 |
| 8372 | cellular component unknown | 9 | 288 | 768 | 1,011 |
