# Supplementary material for: Butyrate-Induced Transcriptional Changes in Human Colonic Mucosa
Source: PLoS One. 2009 Aug 25;4(8):e6759. doi: 10.1371/journal.pone.0006759 (PMC2727000; doi:10.1371/journal.pone.0006759)
Supplement: Table S2 — This table shows the local mapps, ranked by Z-score (0.07 MB DOC) [file pone.0006759.s002.doc]

S2: Local Mapps, ranked by Z-score

| MAPP Name | Number Changed | Number Measured | Number On MAPP | Z Score |
| --- | --- | --- | --- | --- |
| Hs_Electron_Transport_Chain | 14 | 72 | 105 | 6,46 |
| Hs_Krebs-TCA_Cycle | 6 | 19 | 31 | 5,92 |
| Hs_Proteasome_Degradation | 8 | 36 | 61 | 5,38 |
| Hs_Mitochondrial_fatty_acid_betaoxidation | 4 | 11 | 16 | 5,29 |
| Hs_Oxidative_Stress | 6 | 24 | 28 | 5,06 |
| Hs_Fatty_Acid_Beta_Oxidation_Meta_BiGCaT | 5 | 20 | 32 | 4,62 |
| Hs_Glyoxylate_and_dicarboxylate_metabolism | 2 | 5 | 59 | 3,97 |
| Hs_Aminosugars_metabolism | 3 | 14 | 54 | 3,19 |
| Hs_Fatty_Acid_Beta_Oxidation_1_BiGCaT | 3 | 15 | 27 | 3,03 |
| Hs_Valine_leucine_and_isoleucine_degradation | 3 | 16 | 54 | 2,88 |
| Hs_Pyruvate_metabolism | 3 | 17 | 84 | 2,75 |
| Hs_Glycogen_Metabolism | 4 | 30 | 36 | 2,48 |
| Hs_Selenoamino_acid_metabolism | 2 | 11 | 39 | 2,30 |
| Hs_Nucleotide_Metabolism | 2 | 12 | 17 | 2,14 |
| Hs_Ribosomal_Proteins | 4 | 42 | 88 | 1,71 |
| Hs_G13_Signaling_Pathway | 3 | 28 | 37 | 1,70 |
| Hs_Fatty_acid_metabolism | 3 | 28 | 80 | 1,70 |
| Hs_IL-1_NetPath_13 | 3 | 29 | 38 | 1,64 |
| Hs_S1P_Signaling | 2 | 17 | 25 | 1,54 |
| Hs_B_Cell_Receptor_NetPath_12 | 8 | 113 | 158 | 1,52 |
| Hs_Glycolysis_and_Gluconeogenesis | 3 | 32 | 44 | 1,45 |
| Hs_Butanoate_metabolism | 2 | 20 | 75 | 1,28 |
| Hs_Lysine_degradation | 2 | 21 | 75 | 1,20 |
| Hs_Purine_metabolism | 4 | 54 | 181 | 1,16 |
| Hs_IL-3_NetPath_15 | 5 | 74 | 101 | 1,08 |
