# Supplementary material for: Butyrate-Induced Transcriptional Changes in Human Colonic Mucosa
Source: PLoS One. 2009 Aug 25;4(8):e6759. doi: 10.1371/journal.pone.0006759 (PMC2727000; doi:10.1371/journal.pone.0006759)
Supplement: Statistics S1 — This supporting information describes in more detail, the statistics that were performed on the data (0.03 MB DOC) [file pone.0006759.s004.doc]

***Statistics S1***

*Microarray analysis*

The chip description file (CDF) used for the analysis was an update created and freely distributed by the microarray lab of the university of Michigan [10, 11] based on UniGenes (version 8). This resulted in the analysis of 18027 gene-transcripts out of the 54613 commonly obtained using the Human Genome U133 Plus 2.0 CDF provided by Affymetrix.

All gene-transcripts were analysed using a multivariate Gaussian linear regression ( where is the mean, is the covariance matrix , is the variance, and as both the extra component of variance across subjects and the common covariance among responses on the same subject) including the hybridization and labeling spikes, the hybridization day, and a random effect. The inference criterion used for comparing the models is their ability to predict the observed data, i.e. models are compared directly through their minimized minus log-likelihood. When the numbers of parameters in models differ, they are penalized by adding the number of estimated parameters, a form of the Akaike information criterion (AIC) [1].

For each gene, a model containing the relevant covariates mentioned above () was fitted in order to obtain a reference AIC. Then a model containing the treatment group was fitted (). The gene under consideration was found to be differentially expressed if the AIC of this second model decreased compared to the model not containing the treatment.
